# Exploring Virtual Reality (VR) for Memory Enhancement in Alzheimer's Patients

**DOI:** 10.1002/alz70855_097836

**Published:** 2025-12-23

**Authors:** João Vitor Bittencourt Venera

**Affiliations:** ^1^ UNIFEBE, Balneário Camboriú, Santa Catarina, Brazil

## Abstract

**Background:**

Alzheimer's disease (AD) leads to progressive cognitive decline, significantly impairing memory and daily functioning. Traditional therapeutic approaches have had limited success in halting or reversing these deficits. Recent advancements in immersive virtual reality (iVR) offer innovative avenues for cognitive assessment and rehabilitation in AD patients. iVR provides interactive, simulated environments that can be tailored for therapeutic interventions, potentially enhancing cognitive functions and quality of life.

**Method:**

A systematic review was conducted, analyzing studies that employed iVR in the assessment and treatment of individuals with AD or mild cognitive impairment (MCI). Databases searched included Medline, PsycINFO, Embase, CINAHL, and Web of Science. The review focused on the acceptability, feasibility, and efficacy of iVR interventions, as well as their correlation with neuroanatomical substrates and existing screening methods.

**Result:**

Nine (9) studies met the inclusion criteria. Participants with MCI and AD tolerated iVR well during assessment and treatment tasks. One study demonstrated that iVR could detect prodromal AD, correlating with specific neuroanatomical changes. Two studies indicated that iVR accurately differentiated AD patients from healthy controls, though these lacked robust control measures. Regarding treatment, a small validation study and two longitudinal case studies reported positive participant feedback on iVR cognitive training; however, consistent cognitive benefits were not observed.

**Conclusion:**

iVR emerges as a promising tool for the assessment of older adults and individuals with AD, particularly when integrated with theoretical models of neurodegeneration and existing screening methods. While iVR shows potential in cognitive training, its therapeutic efficacy remains inconclusive. Further randomized controlled trials involving clinical populations are necessary to validate iVR's effectiveness in AD assessment and to clarify its role in cognitive rehabilitation beyond current applications in physical therapy.

